# Sociodemographic, HIV-Related Characteristics, and Health Care Factors as Predictors of Self-Reported Vaccination Coverage in a Nationwide Sample of People Aging with HIV in Germany

**DOI:** 10.3390/ijerph18094901

**Published:** 2021-05-04

**Authors:** Jochen Drewes, Phil C. Langer, Jennifer Ebert, Dieter Kleiber, Burkhard Gusy

**Affiliations:** 1 Public Health: Prevention and Psychosocial Health Research, Freie Universität Berlin, 14195 Berlin, Germany; j.ebert123dabei@googlemail.com (J.E.); dieter.kleiber@fu-berlin.de (D.K.); burkhard.gusy@fu-berlin.de (B.G.); 2 Department of Social Psychology, International Psychoanalytic University, 10555 Berlin, Germany; phil.langer@ipu-berlin.de

**Keywords:** vaccination, vaccination coverage, HIV, AIDS, elderly

## Abstract

Preventing infectious diseases through vaccination becomes more significant among the growing population of people aging with HIV. Coverage rates for vaccinations and factors associated with vaccination utilization among this population in Germany are unknown. We assessed the coverage of eight recommended vaccinations in a certain time frame in our convenience sample of 903 people living with HIV aged 50 years and older. We analysed coverage rates and used bivariate and multiple linear regression analyses to identify factors associated with number of reported vaccinations. Coverage rates in our sample ranged between 51.0% for meningococcus disease and 84.6% for the triple vaccination against tetanus, diphtheria, and pertussis. All rates were higher compared to the German general population. Seven factors were related to the number of vaccinations in multiple regression analysis: sexual orientation, education, relationship status, CD4 count, time since last visit to HIV specialist, type of HIV specialist, and distance to HIV specialist. Vaccination coverage among people aging with HIV in Germany is high, but not optimal. To improve vaccination uptake, strengthened efforts need to be focused on female and heterosexual male patients, socioeconomically disadvantaged patients, and patients with barriers to access regular HIV care.

## 1. Introduction

In the growing population of people aging with HIV (PAWH), the prevention and management of comorbidities become more and more significant. PAWH have a higher overall comorbidity burden [1] and a higher risk for more severe courses of comorbid diseases [2,3]—some of these being preventable by vaccination.

Vaccines are in general considered safe and effective in people living with HIV (PLWH); exceptions refer to attenuated vaccines [2,4]. To acknowledge this situation and to increase vaccination coverage among PLWH, national guidelines regarding vaccinations in this population were issued in several countries [2], including Germany [5]. Officially recommended vaccinations are free of cost for the patient in Germany, except vaccinations needed due to travelling. For PAWH, vaccinations against hepatitis A, hepatitis B, tetanus, diphtheria, pertussis, pneumococcal infection, meningococcal infection, and influenza are most relevant, with some of them being booster vaccinations. Other, now recommended, vaccinations against human papillomavirus and herpes zoster were not available or recommended at the time of our study.

While vaccinations are highly recommended to prevent these infectious diseases in PLWH, international studies suggest that vaccination rates in this population are far from perfect [3]. It is difficult to infer tendencies from published studies, because vaccination rates differ vastly across studies due to different study designs, modes of data collection, and study populations. Furthermore, differences in vaccine characteristics and health care systems account for the vast differences between reported coverage rates. For example, coverage rate ranges between 18% [6] and 77% [7] have been reported for hepatitis B, the vaccination with the most published data available. The only nationally representative study on hepatitis B coverage in this population was conducted in the United States, and it found that 53% of PLWH were protected against hepatitis B, either by vaccination or natural immunity [8]. The beforementioned 77% rate reported by a Brazilian study did not include patients with natural immunity, making studies even more difficult to compare. Similarly, large differences in coverage rates were also reported for the influenza vaccination; coverage rates range from 7% in newly diagnosed PLWH [9] to 64% among middle aged and older HIV patients [10].

Some studies reported on factors related to vaccination coverage among PLWH. While these results are also inconsistent and difficult to compare due to differences in vaccination type, study design, and population, it can be observed that factors related to vaccination coverage among PLWH are largely the same previously identified in general population samples, such as age [11], gender [12], and socioeconomic status, operationalized via education, income or insurance status [9,12,13,14,15]. One study identified vaccination hesitancy as a predictor of vaccination uptake among PLWH triggered by unfavorable attitudes towards vaccination in general and towards the side effects of vaccines [11]. Moreover, HIV-specific characteristics were related to vaccination coverage in this population. The best evidence exists for CD4 T-cell number (CD4 count) which was related to vaccination in several studies [10,12,13,16], but also duration of HIV infection [12], an AIDS diagnosis [10], missed clinic appointments [16], and an experienced physician [12] or a physician with a high HIV case load [17] were associated with the uptake of at least single vaccines in this population. Furthermore, within PLWH populations, men having sex with men (MSM) are reported to have better vaccination coverage than non-MSM individuals [12,16].

### Present Study

Utilization rates for officially recommended vaccinations among PLWH are unknown in Germany. With the present study, our aim is to describe self-reported vaccination rates in a large sample of community-dwelling PAWH in Germany. We are further analyzing which sociodemographic, HIV-related, and healthcare-related factors correlate with vaccination coverage.

## 2. Materials and Methods

### 2.1. Study Design

The study ‘50plushiv: Psychosocial aspects of aging with HIV/AIDS in Germany’ was a cross-sectional, exploratory study with a quantitative and a qualitative study arm to describe the health, living conditions, and needs of people aging with HIV and AIDS in Germany conducted in 2013–2014. This paper uses only the quantitative data. Eligible participants were all people diagnosed with HIV, 50 years old or older. The questionnaire was provided as an online questionnaire and as a paper pencil questionnaire (for more information, see [18]). All information obtained from the participants is self-reported. All participants provided written informed consent. The study was approved by the Freie Universität Berlin ethics committee.

### 2.2. Participants

A total of 907 people who met the eligibility criteria completed either the paper or the online questionnaire. We eliminated 3 subjects from our analyses regarding vaccination coverage, because they showed missing values for all vaccination items; thus, our final sample consisted of 904 subjects.

### 2.3. Primary Outcome Variable

We assessed subjects’ participation in eight vaccinations in a certain period: vaccination against hepatitis A (lifetime), hepatitis B (lifetime), pneumococcal disease (last 5 years), tetanus (last 10 years), diphtheria (last 10 years), pertussis (last 10 years), meningococcal disease (last 5 years), and influenza (last 12 months). Vaccinations against tetanus, diphtheria, and pertussis were combined in one item, because these vaccinations are administered as a combination vaccination. Response options were ‘yes’, ‘no’, and ‘don’t know’, except for the vaccination against hepatitis A, for which we added the option ‘already infected’ to assess immunity following a previous infection. For the vaccination against hepatitis B, a similar option was not provided as lifetime infection with hepatitis B was assessed elsewhere in our questionnaire. Participants who reported a hepatitis B infection were classified as ‘already infected’ for the hepatitis B vaccination variable retrospectively.

We calculated our primary outcome variable ‘number of vaccinations’ by adding the value 1 for any vaccination the participant reported in the appropriate time frame. For a lifetime history of a hepatitis A or B infection, the value 0.5 was added to acknowledge that these participants did not have the equal chance for a hepatitis A or B vaccination in their life course. The variable ‘full coverage of vaccinations’ which we only used for descriptive analyses was built by adding ‘yes’ answers across all six vaccination items without considering ‘don’t know’ or ‘already infected’ answers. Participants who reached a score of six were categorized as having had full coverage of vaccinations.

### 2.4. Sociodemographic and Health Care Variables

We used various sociodemographic variables for descriptive purposes and as predictors of vaccination and cancer screening coverage, as age, gender (male vs. female), sexual orientation (heterosexual vs. not heterosexual), size of place of residence (less than 500,000 vs. more than 500,000), relationship status (single vs. in relationship), education (10 years and less vs. more than 10 years), and socioeconomic status (SES; combined metric variable; see [18,19] for details). We further used HIV-related variables such as duration of HIV infection, CD4 count (less than 200 vs. more than 200) and HIV RNA level (viral load; undetectable vs. detectable), an AIDS diagnosis, and late HIV diagnosis (see [18] for details), and health care variables such as type of HIV specialist provider (private practice vs. hospital/clinic), distance to HIV specialist (less than 10 km vs. more than 10 km), last visit to HIV specialist (within 3 months vs. longer than 3 months), type of insurance (public vs. private), and whether the patient was regularly consulting a general practitioner apart from the HIV specialist (no vs. yes).

### 2.5. Statistical Analyses

All analyses were conducted using IBM SPSS version 26 (IBM SPSS Statistics, Chicago, IL, USA). Nominal and ordinal variables with more than two attributes were dichotomized. We tested bivariate associations between sociodemographic, HIV-related and health care variables, and our primary outcome variable using simple regression analyses. A multivariate regression model was calculated using all variables that reached a *p*-value < 0.10. Due to a relatively high percentages of missing values in certain variables, especially CD4 count and SES (see Table 1), we chose pairwise deletion of cases with missing values in the multivariate regression model. While education and socioeconomic status were both significant predictors of vaccination in bivariate regression analyses, we chose the variable with the higher β-value for the multivariate regression model, because the original education variable was used among others to calculate the socioeconomic status variable and both variables correlated highly.

## 3. Results

Our sample was predominantly male (87%), of which 704 identified as bi- or homosexual (90% of male participants, 78% of all participants). The mean age was 57 years, with the majority of 71% being between 50 and 59 years old. Subjects were living on average 17 years with HIV (time since diagnosis), and 90% reported an undetectable viral load (see Table 1). Table 1 also shows the average number of vaccinations for each subgroup.

Self-reported utilization rates for each vaccination can be found in Table 2. Rates range between 51.0% for meningococcus disease and 84.6% for the triple vaccination against tetanus, diphtheria, and pertussis. Table 2 also shows the missing values due to nonresponding or ‘don’t know’ responses. For hepatitis A and B infection, the table also shows the number of participants indicating they were already infected with each infection (see Methods section for further information). On average, participants reported 3.8 vaccinations, but 5% reported completion of none of the eight vaccinations, and only 20% reported completion of all vaccinations.

Table 3 shows the vaccination rates for each vaccination for three age groups and a comparison with the national representative data from the general population for hepatitis A, hepatitis B, influenza, and meningococcal vaccination [20]. The rates in our sample are for every age group and vaccine combination much higher than in the general population sample, up to 51 times higher for the meningococcal vaccination in the 70–79 years age group.

### Regression Analyses

In bivariate analyses, gender, sexual orientation, SES, education, size of place of residence, relationship status, HIV viral load, CD4 count, time since last visit to HIV provider, type of HIV provider, distance to HIV provider, and presence of an additional general practitioner were all statistically significantly related to the number of reported vaccinations (see Table 4). In multivariate analyses, seven of these variables were still significantly related with the number of vaccinations: sexual orientation (*p* = 0.011), education (*p* = 0.013), relationship status (*p* = 0.027), CD4 count (*p* = 0.001), time since last visit to HIV specialist (*p* = 0.046), type of HIV specialist (*p* = 0.047), and distance to HIV specialist (*p* = 0.033).

## 4. Discussion

Our study is the first published report of coverage of vaccinations in people living with HIV in Germany. In our convenience sample of more than 900 HIV-positive individuals aged 50 years or more, we found rather high rates of accomplished vaccinations; however, utilization rates remained far from optimal. Based on self-reports, each vaccination was accomplished by at least half of our participants, but only one in five reported full adherence to vaccination guidelines with accomplishment of all eight vaccinations that were assessed in our study. While utilization rates in our sample were not optimal, utilization for each vaccination was higher than in most international studies among PLWH and in the German general population. We found several factors associated with vaccination utilization, with the strongest association for CD4 count, sexual orientation, distance to HIV provider, and SES.

The lowest vaccination rate of 51% was found for the meningococcus vaccination, which showed the largest differences compared to the general population. While meningococcal vaccination is recommended for PLWH in Germany, it was also added to our questionnaire, because of an outbreak of invasive meningococcal serogroup C disease among MSM in Berlin prior to our study which resulted in an official vaccination recommendation for MSM living in Berlin [21]. A recommendation for the general population was not issued before 2006 and did not include a recommendation for adults. This can explain the large differences in vaccination rates between both populations. Further, the rate of meningococcal vaccination in our sample is consistent with a self-reported vaccination rate of 70% among HIV-infected MSM in Berlin, while non-HIV-infected MSM in Berlin only had a vaccination rate of 13% reported in an evaluation of the official recommendation [21].

The self-reported utilization rates for each of the other vaccinations are higher, with at least two thirds of our participants reporting receiving the respective vaccination in the recommended time frame. Where comparable data was available, all our rates were on the higher end of rates in other samples of PLWH, and higher than in the general German population.

Hepatitis B vaccination was reported by 75% of our participants without natural immunity after infection; taking these participants into account, 81% of our sample of PAWH were protected against hepatitis B infection either by vaccination or previous infection. While international studies report coverage rates for hepatitis B vaccination among PLWH as low as 37%, a Brazilian study found a similar rate of 77% without natural immunity. Moreover, a large internet study among MSM in Europe showed that 62% of participating MSM in Germany reported receiving all three doses of the hepatitis B vaccination [22]. A study using blood samples from HIV-positive MSM in Germany showed a differentiated picture: while 80% of participants between 45 and 54 years and 71% of participants older than 54 years were protected against hepatitis B infection, only 32% vs. 23% in each age group were effectively vaccinated [23]. While we reported similar rates of patients being susceptible to hepatitis B infection, the proportions between patients being protected by previous infection and patients being protected by effective vaccination were almost inverted across both studies. These discrepancies can be explained in several ways, by overreporting of vaccination and underreporting of infection in our sample (which will be discussed further in the ‘limits’ section), by miscategorization of blood samples (e.g., through weak immune responses to vaccination in PLWH) in the other study or by differences in sample composition, because both studies relied on convenience samples, did target slightly different populations, and employed different ways of recruiting participants.

Utilization of influenza vaccination in the previous 12 months was high in our sample and reached 77% the World Health Organization’s goal of 75% coverage in older and immunocompromised populations [24]. Discrepancies with the general population are high and particularly high in the 50 to 59 years age group, because recommendations for influenza vaccination in the general population target only individuals aged 60 years and older [20]. Influenza coverage rates in other samples of PLWH are lower than in our sample, among newly diagnosed PLWH, coverage was as low as 7% [9], but in older populations of PLWH, the rates were similarly high, at 64%. While influenza vaccination is recommended for all PLWH regardless of age, coverage rates are, similarly to the general population, highest in older populations, a result reported by several studies [25,26,27].

Several variables were associated with vaccination utilization in bivariate analyses, and seven of them were statistically significant in multivariate analyses; being homosexual or bisexual and having a CD4 count of more than 200 cells had the biggest positive influence on vaccination coverage. While our sample consisted predominantly of homosexual men, the MSM-specific vaccination recommendation for meningococcal disease could be an explanation for the strong influence of sexual orientation on vaccination coverage; on the other hand, sexual orientation was also a predictor of vaccination coverage in several international studies [12,16,27]. Table 1 also shows that, not bi-/homosexuality, but male bi-/homosexuality, is associated with more reported vaccinations. In fact, bi- and homosexual women reported fewer vaccinations than heterosexual women, making bi-/homosexuality for women a disabling factor regarding vaccination coverage. CD4 count was a predictor of vaccination coverage in several international studies as well, probably reflecting uncertainty about vaccinating immunocompromised patients among HIV providers. While a CD4 count below 200 cells defines an immunocompromised HIV patient, vaccination guidelines only advise against attenuated vaccines for patients with a CD4 count below 200 cells. The vaccinations that our study was focusing on are all also recommended for these immunocompromised patients [2,5].

Among the remaining predictors are education as a marker of socioeconomic status, a variable that was reported as a predictor of vaccination coverage in many studies with PLWH and general population samples [9,12,13,14,15,20], and three healthcare related variables. Patients who lived more than 10 km away from their HIV specialist and patients who did not adhere to regular visits to the HIV specialist, reported less vaccinations, while patients who visited a private practice compared to a hospital or outpatient clinic reported less vaccinations. Distance and adherence obviously affect the frequency with which a provider is seen, and more visits to the HIV provider seem to enable more vaccination opportunities. The better vaccination coverage among patients visiting a private practice might be explained by a different or stronger patient–doctor relationship in private practices. An unexpected finding was the relationship between relationship status and vaccination coverage. The association may be explained by the higher amount of social support experienced by patients in relationship; social support is known to influence uptake of healthy behaviours, including vaccination [28].

### Limits

Various factors limit the validity and generalizability of our results. We were not able, for several reasons, to realize a probability sample of PAWH in Germany. Thus, our results regarding vaccination and cancer screening rates cannot be generalized to the population of PAWH in Germany. While real probability samples of this hidden population are almost impossible to realize, our sample is, by far, the biggest and most diverse sample of PAWH in Germany in the published literature.

Our study employed a cross-sectional study design; thus, statistically, we cannot infer causality between our predictors and primary outcome variables. Our results, although in accordance with other studies in this population, should thus be interpreted with caution and need confirmation by longitudinal studies.

A major difference of our study compared to most other similar studies is the reliance on self-report in our study. Self-report, in contrast to checking medical records, allows for the realization of larger samples in a cost-economic way, and does not restrict the sample to one or more medical practices or clinics. However, while the validity of self-reports regarding medical procedures is contested, a study among elderly outpatients found a moderate agreement between medical records and self-report due to overreporting [29].

While the overreporting could explain the large differences in vaccination and cancer screening coverage between our sample and the German general population sample (see Table 3), it must be noted that this study also relied on self-reports if vaccination certificates were not available at the interview [20]. Our comparisons between PAWH and the general population are still to be interpreted with caution. The larger general population sample is representative for the German population and differs from our study in terms of study design and in mode of data collection. Being a convenience sample, our sample will most likely show a significantly different composition biased for several characteristics which are known to influence coverage, such as socioeconomic status (see [18] for a comparison of our sample with nationally representative data regarding socioeconomic status).

Finally, we were unable to employ all variables that are known to be important predictors of vaccination and cancer screening uptake, such as attitudes towards these procedures, or receipt of a provider recommendation for the procedures, in our extensive questionnaire due to space and time limits. Our regression model is thus explorative and regression weights are to be interpreted with caution—however, in the absence of a theoretical approach to explain vaccination uptake, all models are inevitably explorative.

## 5. Conclusions

This study reported, for the first time, vaccination rates among PAWH in Germany and revealed high rates of self-reported coverage rates compared to international samples of PLWH and the older general population in Germany. While overreporting and a biased convenience sample may explain exaggerated coverage rates, we conclude, from our study, that vaccination rates among German PAWH are better than in the general population and among PLWH in other countries. Nonetheless, coverage rates are not optimal. Large parts of our sample were not adequately immunized against meningococcal infection, influenza, pneumococcal infection, and hepatitis A, and only two in five participants reported a full or almost full coverage of all assessed vaccinations. To improve vaccination coverage in this population, our study points to several indications. HIV providers need more information on the safety, efficacy, and importance of vaccination among PAWH, especially on the safety of non-attenuated vaccines in immunocompromised patients. Hospitals and clinics with HIV outpatients need to improve vaccination rates in this population. HIV patients who need to be in the focus of strengthened efforts are female and heterosexual male patients, socioeconomically disadvantaged patients, and patients with barriers to access regular HIV care.

## Figures and Tables

**Table 1:** Sample characteristics and number of self-reported vaccinations for each subgroup (n = 904).

	N (%) or Mean (SD)	No of Vaccinations Mean (SD)
**Total**	904	3.8 (1.7)
**Age**	57.3 (SD = 6.6)	
50–59 years	645 (71.3%)	3.9 (1.7)
60–69 years	193 (21.3%)	3.7 (1.7)
70 years and older	66 (7.3%)	3.8 (1.6)
**Gender**		
male	783 (86.6%)	3.9 (1.7)
female	117 (12.9%)	3.3 (1.8)
trans	4 (0.4%)	3.6 (0.9)
**Sexual orientation**		
heterosexual men	76 (8.4%)	3.1 (1.7)
homo-/bisexual men	701 (77.7%)	4.0 (1.6)
heterosexual women	108 (12.0%)	3.4 (1.8)
homo-/bisexual women	8 (0.9%)	2.9 (1.7)
other	9 (1.0%)	3.2 (1.8)
**Education**		
10 years or less	487 (55.2%)	3.6 (1.7)
more than 10 years	396 (44.8%)	4.1 (1.6)
**Socioeconomic status**	12.4 (SD = 4.0)	
low ^1^	124 (14.7%)	3.5 (1.7)
middle ^1^	408 (48.5%)	3.8 (1.7)
high ^1^	309 (36.7%)	4.1 (1.5)
**Size of place of residence**		
less than 500,000 inhabitants	401 (44.9%)	3.5 (1.7)
more than 500,000 inhabitants	493 (55.1%)	4.1 (1.6)
**Relationship status**		
single	416 (46.2%)	3.7 (1.7)
in relationship	484 (53.8%)	4.0 (1.6)
**Duration of HIV-infection**	16.8 (SD = 8.6)	
1–5 years	104 (11.5%)	3.7 (1.8)
6–10 years	151 (16.7%)	4.1 (1.5)
11–20 years	313 (34.7%)	3.9 (1.6)
21 years and longer	334 (37.0%)	3.6 (1.8)
**HIV-1 RNA**		
detectable	88 (10.2%)	3.5 (1.8)
undetectable	778 (89.8%)	3.9 (1.6)
**CD4 count**		
less than 200 cells	27 (3.5%)	2.6 (1.4)
200 cells and more	734 (96.5%)	3.9 (1.6)
**Last visit to HIV specialist**		
less than three months	862 (96.3%)	3.9 (1.7)
more than three months	33 (3.7%)	3.2 (2.0)
**AIDS diagnosis**		
yes	299 (34.9%)	3.8 (1.8)
no	558 (65.1%)	3.9 (1.6)
**Late diagnosis**		
yes	269 (30.1%)	3.8 (1.6)
no	625 (69.9%)	3.9 (1.7)
**Type of HIV specialist**		
private practice	702 (79.3%)	4.0 (1.6)
clinic/hospital	183 (20.7%)	3.5 (1.8)
**Distance to HIV specialist**		
less than 10 km	577 (64.0%)	4.0 (1.6)
more than 10 km	325 (36.0%)	3.5 (1.7)
**Presence of additional general practitioner**		
yes	328 (36.6%)	3.6 (1.7)
no	567 (63.4%)	4.0 (1.7)
**Type of health insurance: private**		
public	738 (84.0%)	3.8 (1.7)
private	141 (16.0%)	4.0 (1.6)

^1^ low = 0–20% of general population, middle = 20–80% of general population; high = 80–100% of general population (see [18] for more information).

**Table 2:** Self-reported coverage of vaccinations among people aging with HIV in Germany (2013, n = 904).

	Yes	No	Already Infected	Don’t Know	Missing
	n (%)	n (%)	n	n	n
Hepatitis A	534 (69.3%)	237 (30.7%)	29	89	18
Protected ^1^	563 (70.4%)	237 (29.6%)	-	89	18
Hepatitis B	481 (75.3%)	158 (24.7%)	195	61	12
Protected ^1^	676 (81.1%)	158 (18.9%)	-	61	12
Pneumococcal infection	545 (66.3%)	277 (33.7%)	-	76	9
Tetanus, diphtheria, pertussis	730 (84.6%)	133 (15.4%)	-	35	9
Meningococcal infection	368 (51.0%)	354 (49.0%)	-	171	14
Influenza	693 (77.1%)	206 (22.9%)	-	4	4
**Number of reported vaccinations**	M = 3.8 (1.7)				
none	45 (5.0%)				
one	69 (7.6%)				
two	102 (11.3%)				
three	138 (15.3%)				
four	196 (21.6%)				
five	174 (19.2%)				
full coverage	180 (19.9%)				

^1^ ‘Yes’ and ‘already infected’responses added.

**Table 3:** Self-reported coverage of vaccinations among people aging with HIV in Germany (50plushiv) and a nationally representative sample of the German general population (DEGS1 ^1^) [20] by age group.

	50Plushiv (N = 904)			DEGS ^1^ (N = 7988)		
	50–59	60–69	70–79	50–59	60–69	70–79
Hepatitis A	72.0%	59.6%	69.8%	23.3%	20.6%	11.9%
Hepatitis B	78.0%	65.4%	77.8%	22.7%	16.4%	9.5%
Influenza	75.7%	77.6%	89.1%	44.0%	63.8%	68.3%
Meningococcal infection	53.2%	47.1%	40.9%	1.8%	2.0%	0.8%
Tetanus, diphtheria, pertussis	85.9%	81.8%	81.0%	- ^2^	- ^2^	- ^2^
Pneumococcal infection	65.6%	69.4%	62.7%	- ^2^	- ^2^	- ^2^

^1^ DEGS—Studie zur Gesundheit Erwachsener in Deutschland (The German Health Interview and Examination Survey for Adults); ^2^ no comparable data available in source.

**Table 4:** Factors associated with vaccination coverage among people aging with HIV in Germany (2013, n = 904).

		Bivariate Regression		Multivariate Regression	
	n	β	*p*	β	*p*
Age	904	−0.058	0.079		
Gender: female	900	**−0.113**	**0.001**	0.006	0.900
Sexual orientation: not heterosexual	895	**0.180**	**0.000**	**0.127**	**0.011**
Socioeconomic status	841	**0.125**	**0.000**		
Education: more than 10 years	883	**0.153**	**0.000**	**0.091**	**0.013**
Size of place of residence: more than 500,000 inhabitants	894	**0.170**	**0.000**	0.053	0.238
Relationship status: in relationship	900	**0.103**	**0.002**	**0.079**	**0.027**
Duration of HIV infection	902	−0.052	0.118		
HIV−RNA: undetectable	866	**−0.072**	**0.033**	−0.049	0.169
CD4 count: more than 200	761	**0.146**	**0.000**	**0.121**	**0.001**
Last visit to HIV specialist: more than three months	895	**−0.073**	**0.028**	**−0.070**	**0.046**
AIDS diagnosis: yes	857	−0.006	0.855		
Late diagnosis: yes	894	−0.015	0.648		
Type of HIV specialist: private practice	885	**0.116**	**0.001**	**0.075**	**0.047**
Distance to HIV specialist: more than 10 km	902	**−0.155**	**0.000**	**−0.092**	**0.033**
Presence of additional general practitioner: yes	895	**−0.093**	**0.005**	0.005	0.905
Type of health insurance: private	879	0.041	0.223		

## Data Availability

The dataset generated and analysed during the current study is available from the corresponding author on reasonable request.
